# Biological Control of *Verticillium* Wilt on Olive Trees by the Salt-Tolerant Strain *Bacillus velezensis* XT1

**DOI:** 10.3390/microorganisms8071080

**Published:** 2020-07-20

**Authors:** David Castro, Marta Torres, Inmaculada Sampedro, Fernando Martínez-Checa, Borja Torres, Victoria Béjar

**Affiliations:** 1 Xtrem Biotech S.L., European Business Innovation Center, 18100 Granada, Spain; dcastro@xtrembiotech.com (D.C.); btorres@xtrembiotech.com (B.T.); 2 Department of Microbiology, Faculty of Pharmacy, University of Granada, 18071 Granada, Spain; isampedro@ugr.es (I.S.); fmcheca@ugr.es (F.M.-C.); vbejar@ugr.es (V.B.); 3 Biomedical Research Center, Institute of Biotechnology, 18016 Granada, Spain

**Keywords:** biocontrol, antifungal activity, crop protection, *Bacillus velezensis*, *Verticillium dahliae*, *Olea europaea*

## Abstract

*Verticillium* wilt, caused by the pathogen *Verticillium dahliae*, is extremely devastating to olive trees (*Olea europea*). Currently, no successful control measure is available against it. The objective of this work was to evaluate the antifungal activity of *Bacillus velezensis* XT1, a well-characterized salt-tolerant biocontrol strain, against the highly virulent defoliating *V. dahliae* V024. In vitro, strain XT1 showed to reduce fungal mycelium from 34 to 100%, depending on if the assay was conducted with the supernatant, volatile compounds, lipopeptides or whole bacterial culture. In preventive treatments, when applied directly on young olive trees, it reduced *Verticillium* incidence rate and percentage of severity by 54 and ~80%, respectively. It increased polyphenol oxidase (PPO) activity by 395%, indicating an enhancement of disease resistance in plant tissues, and it decreased by 20.2% the number of fungal microsclerotia in soil. In adult infected trees, palliative inoculation of strain XT1 in the soil resulted in a reduction in *Verticillium* symptom severity by ~63%. Strain XT1 is biosafe, stable in soil and able to colonize olive roots endophytically. All the traits described above make *B. velezensis* XT1 a promising alternative to be used in agriculture for the management of *Verticillium* wilt.

## 1. Introduction

*Verticillium* wilt, caused by *V. dahliae,* is a soil-borne fungal disease. First described in Italy [1], it affects around 400 plant species, ranging from herbaceous annuals to woody perennials, such as cotton, tomato, alfalfa, almond, pistachio, and peach [2,3]. Monocotyledonous plants are not affected, although they may act as asymptomatic reservoirs of the fungi [4], and only dicotyledonous plants can be attacked by the disease. Amongst the latter, *V. dahliae* is extremely devastating to olive trees (*Olea europaea*) and is currently considered the main soil-borne disease threatening olive production. Infection by *Verticillium* not only affects the volume of production, but also the quality and properties of the oil produced [5]. It affects all the olive-growing regions around the world, especially the Mediterranean area [6,7], and causes severe economic losses due to plant death [6]. Notably, *Verticillium* wilt is a serious threat to the olive industry in Spain, the world’s largest producer of olive oil by volume with more than half of world production [8]. In particular, the disease is a major concern in the Andalusia region of southern Spain, which produces 83% of total olive oil in Spain [9]. In this region, the mean disease incidence is 20.4% [10], although this percentage could be underestimated due to the absence of more recent data. 

In its life cycle, *V. dahliae* [11] forms conidiospores and microsclerotia as resistant structures. The latter are darkly melanized mycelial cells which can persist in soil and dead material for more than 10 years, being the primary long-term survival structures. Once they germinate, the hyphae are attracted to the plant rhizosphere by a nutrient gradient and coat the plant roots [12]. Both the hyphae and the conidiospores can invade the healthy and damaged plant tissues and colonize the plant vascular system very rapidly [13]. As a result, water and nutrient balance are altered and the typical symptoms of *Verticillium* wilt, such as chlorosis or defoliation, appear [14]. 

Nowadays, no successful control measure of *Verticillium* wilt is available. The control of the disease is primarily done by preventive before-planting measures. These include the protection of plant material from *V. dahliae* infection during plant propagation and/or at transplanting using antagonistic biocontrol agents [11,15,16]. Disease management is hindered by the absence of effective fungicides, which are ineffective once the fungus has reached the plant vascular system, and the restriction of preventive chemical fumigants [17]. This, together with the demand of the society to render the agriculture industry more sustainable while maintaining a healthy environment, is pushing to search new strategies to grow crops with reduced agrochemicals, for instance using biological control agents as an alternative. 

Biological control (or biocontrol) agents are microorganisms which colonize the plant rhizosphere and/or phylosphere, control pathogens, and act as fertilizers by several mechanisms [18,19]. Examples of these mechanisms are competition for ecological niches or production of volatile compounds and lipopeptide biosurfactants, which improve plant health by diminishing root-colonizing phytopathogens [20,21]. A number of these mechanisms lead to an increase in plant resistance to infections, known as induced systemic resistance [22]. Some of the desirable characteristics looked for in bacteria to be used as biocontrol tools against *Verticillium* are the reduction in the survival or germination of microsclerotia, the colonization of the host plant, the competition with the pathogen or the induction of resistance responses in the plant. Such characteristics should be evaluated in conditions which are similar to the field situation, in order to increase the chance of successful use in practice [23].

To date, selected isolates of several fungal species have been described as biological control agents against *Verticillium.* For instance, *Trichoderma asperellum* [24] and *Fusarium oxysporum* [25] have been identified as potential tools to fight *Verticillium* wilt. Concerning bacterial biocontrol agents, *Serratia plymuthica* [26], *Paenibacillus alvei* [27], and *Pseudomonas fluorescens* [28] have proved to be effective against *V. dahliae* in plant experiments. Regarding the members of the genus *Bacillus,* frequently used in agricultural systems as biocontrol tools, *B. subtilis* [29] and *B. velezensis* [30] have recently been reported to successfully reduce *Verticillium* wilt in planta. However, none of the biocontrol agents previously described have been isolated from saline environments. Soil salinization is a problematic issue that reduces agricultural productivity and is exacerbating with climate change. In recent years, it has been shown that salinity increases plant colonization and disease severity by *V. dahliae* [31,32] and reduces the activity of some *Verticillium* biocontrol agents [33]. Therefore, application of salt-tolerant biocontrol and plant growth promotion microorganisms seems to be a promising strategy to fight *Verticillium* wilt.

The objective of this study was to assess the antifungal activity of *B. velezensis* XT1, a well-characterized salt-tolerant biocontrol strain previously isolated in Andalusia (Spain) [34], against the fungal pathogen *V. dahliae* V024, a defoliating pathotype characterized as highly virulent [35]. The preventive and palliative application of *B. velezensis* XT1 on olive trees affected by *Verticillium* wilt was evaluated initially under non-saline conditions in greenhouse and field experiments.

## 2. Materials and Methods

### 2.1. Strains and Routine Culture Conditions

*B. velezensis* XT1 (CECT 8661) was grown in tryptic soy broth (TSB) at 30 °C and 100-rpm rotary shaking and maintained on tryptic soy agar (TSA). The reference strains *Escherichia coli* MC4100 (CGSC 6152), *E. coli* OP50 (Carolina Biological, Burlington, USA), *Pseudomonas aeruginosa* PAO1 (CECT 4122), *Pseudomonas putida* KT2440 (ATCC 47054), and *Burkholderia cepacia* CC-Al74 (provided by the Department of Microbiology at the University of Granada, Spain) were also grown in TSB at 30 °C and 100-rpm rotary shaking and maintained on TSA. *Aliivibrio* (formerly *Vibrio*) *fischeri* NRRL B-11177 (ATCC 49387) was grown in marine broth (MB) at 30 °C and 100-rpm rotary shaking. *V. dahliae* V024, obtained from the collection of the Department of Agronomy at the University of Córdoba (Spain), was cultivated in potato dextrose broth (PDB) at 25 °C with 100-rpm rotary shaking and maintained on potato dextrose agar (PDA). *Caenorhabditis elegans* was maintained in nematode growth media (NGM) [36]. 

### 2.2. Evaluation of the Toxicity of B. velezensis XT1

The biosafety of *B. velezensis* XT1 was evaluated with a set of experiments [37] to assess its potential impact on microbial viability (*E. coli* MC4100 sensitivity test), microbial metabolism (Microtox assay in *A. fischeri* NRRL B-11177), and on the survival of soil nematodes (*C. elegans* N2 bioassay).

#### 2.2.1. *E. coli* MC4100 Sensitivity Assay

The effect of *B. velezensis* XT1 on the survival of *E. coli* MC4100 was evaluated according to reference [37]. Briefly, 500 µL of the filtered supernatant of an overnight culture of strain XT1 in TSB (10^8^ CFU mL^−1^) were mixed with the same volume of a suspension of *E. coli* MC4100 (10^9^ CFU mL^−1^) in M9 buffer (3 g L^−1^ KH_2_PO_4_, 6 g L^−1^ Na_2_HPO_4_, 5 g L^−1^ NaCl, and 1 mL L^−1^ MgSO_4_ 1M; final pH 7.2) and incubated 1.5 h at 25 °C. The mixtures were spread on TSA plates to estimate *E. coli* CFU mL^−1^. The filtered supernatants of overnight cultures (10^8^ CFU mL^−1^) of two biocontrol strains proposed in the literature, *P. putida* KT2440 and *B. cepacia* CC-Al74, and 0.2% (*w*/*v*) copper sulfate, widely used in agriculture, were used for comparison. Non-inoculated TSB was used as a control. The assay was conducted in triplicate.

#### 2.2.2. *A. fischeri* NRRL B-11177 Microbial Metabolism Evaluation 

Microbial metabolism assays were performed according to Johnson (2005) [38] using the supernatant of a 48-h culture of *B. velezensis* XT1 (10^8^ CFU mL^−1^) in Cooper medium [39]. The assay was conducted with the bioluminescent *A. fischeri* NRRL B-11177 (10^8^ CFU mL^−1^) using a Microtox 500 luminescence analyzer (Instrumentación Analítica S.A., Madrid, Spain). Toxicity was expressed as the concentration of *B. velezensis* XT1 supernatant that reduced 50% (EC50) of the initial luminescence of *A. fischeri* after 15 min exposure [40]. Non-inoculated Cooper medium was used as a control. The assay was conducted in triplicate.

#### 2.2.3. *C. elegans* N2 Pathogenicity Assay

The impact of *B. velezensis* XT1 on the survival and viability of soil nematodes was evaluated on the *C. elegans* N2 model organism. The assay was conducted according to Darby et al. [41], with the modifications described by Navas et al. [42]. Briefly, 20 μL of an overnight culture of strain XT1 in TSB (10^8^ CFU mL^−1^) were spread on NGM [36] and incubated 24 h at 30 °C. Then, five adult larvae were added to each plate. *P. aeruginosa* PAO1, a pathogenic strain that causes the death of the nematode [43], was used for comparison. *E. coli* OP50 [44] was used as a control. The volume and concentration (10^8^ CFU mL^−1^) as with strain XT1 were used in the case of *P. aeruginosa* PAO1 and *E. coli* OP50. Plates were incubated at 24 °C for 8 days. The nematodes were examined at 20X magnification and the number of alive individuals was recorded every day. Five replicates were conducted.

The *C. elegans* model was also used to determine the concentration of strain XT1 that was lethal to 50% of the individuals (LC50). The assay was conducted according to Williams and Dusenbery [45]. Briefly, ten adult larvae were transferred with a needle to each well of a 48-well culture plate, filled with 50 μL of a culture of *E. coli* OP50 (10^9^ CFU mL^−1^) and 500 μL of K+ medium [46]. The supernatant of a culture of strain XT1 (10^8^ CFU mL^−1^) in Schaeffer’s-glucose (SG) medium [47] was diluted 1/2, 1/4, 1/8, and 1/50 in liquid NGM and added to the wells up to 1 mL final volume. Plates were incubated at 20 °C for 24 h. The nematodes were examined at 20× magnification and the number of alive individuals was recorded. Copper sulfate, commonly used in agriculture as a fungicide, was used at final concentrations of 0.2% (*w*/*v*) for comparison, and non-treated wells were used as controls. The assay was repeated twice.

### 2.3. In Vitro Evaluation of the Antifungal Activity of B. velezensis XT1 

The antifungal activity of the whole culture, supernatant, crude lipopeptide extract, and volatile compounds produced by *B. velezensis* XT1 were tested in five different types of assays against *V. dahliae* V024.

#### 2.3.1. Assay in Liquid Media

Inhibition of fungal conidiospore germination (and posterior mycelium formation) was evaluated using the supernatant of a liquid culture of strain XT1 [48,49]. Briefly, a 7-day culture of *V. dahliae* was crushed and filtered through an 80-µm pore gauze. This conidiospore suspension was adjusted to a concentration of 10^7^ conidiospores mL^−1^, and was treated with 2.5 µg mL^−1^ benzylpenicillin and 10 µg mL^−1^ streptomycin to avoid bacterial contamination. Strain XT1 was cultivated in nutritive broth (NB) for 72 h at 30 °C (10^8^ CFU mL^−1^) with 100-rpm rotary shaking. The culture was centrifuged at 12,000×g for 15 minutes and the supernatant was filtered through a 0.22 µm-pore membrane filter. A mix of 900 µL of *V. dahliae* conidiospore suspension and 300 µL of the filtered supernatant of strain XT1 were placed in the wells of a 48-well culture plate. The same volume of conidiospore suspension supplemented with 50 µg mL^−1^ cycloheximide was used as a control for fungal growth inhibition, while the conidiospore suspension alone was used as a control for fungal growth. After 30 days of incubation at 25 °C with 100-rpm rotary shaking, the growth of the pathogenic fungi was assessed visually. Assays were carried out in triplicate.

#### 2.3.2. Assay on Solid Media

Mycelium inhibition was measured on solid medium as described by Ji et al. [50] following the modifications by Torres et al. [34]. Ten microliters of an overnight culture of XT1 in TSB (10^8^ CFU mL^−1^) were point-inoculated on the surface of a PDA plate. Then, a 4-mm agar plug of *V. dahliae* mycelium was deposited on the opposite side of the plate. After 20 days of incubation at 25 °C, the mycelium inhibition rate [IR%=(A-B)/A×100] was calculated, considering A as the maximum and B the minimum values of the mycelium radius. Non-inoculated TSB was used as a control. The assay was conducted in triplicate. 

#### 2.3.3. Assay on Modified Solid Media

A modified solid medium was prepared using the supernatant of *B. velezensis* XT1 instead of distilled water to prepare PDA plates. Briefly, a 3-day culture (10^8^ CFU mL^−1^) of strain XT1 in optimal medium for lipopeptide production (MOLP) [51] was centrifuged at 5000×g for 5 min and filtered through a 0.22 µm pore-size membrane filter. The filtered supernatant was used undiluted and diluted to half (with distilled water) to hydrate the PDA powder. Then, pH was adjusted to 5.6 ± 0.2 and the medium was sterilized. Once the modified solid media were ready, a 4-mm plug of *V. dahliae* mycelium was placed in the center of the plates, which were incubated for 20 days at 25 °C. The control consisted of PDA hydrated with distilled water alone. The percentage of mycelium inhibition was calculated by image analysis using ImageJ software [52], measuring the area of fungal growth and comparing it to the control. Three replicates were conducted. 

#### 2.3.4. Volatile Compounds Antifungal Test 

The effect of volatile compounds produced by *B. velezensis* XT1 on *V. dahliae* was tested on bipartite petri dishes. A 4-mm plug of *V. dahliae* mycelium was placed on one half of the dish, containing PDA. On the other half, containing MOLP, 10 µL of an overnight culture of XT1 in TSB (10^8^ CFU mL^−1^) were point-inoculated on the surface of the medium. Non-inoculated TSB was used as a control. Plates were sealed and incubated for 15 days at 25 °C. Fungal growth was assessed by image analysis using ImageJ software. The assay was repeated three times. 

#### 2.3.5. Crude Lipopeptide Extract Antifungal Test 

The effect of the lipopeptides produced by *B. velezensis* XT1 on fungal conidiospore germination and mycelium formation was evaluated using a crude lipopeptide extract of strain XT1 instead of water to prepare the medium. The lipopeptides produced by strain XT1 were previously identified as four different types of surfactin, three bacillomycins D and fengycin A and B [49]. Briefly, lipopeptides were extracted from a 3-day culture of strain XT1 on MOLP (10^8^ CFU mL^−1^), according to Yazgan et al. [53]. The culture supernatant of strain XT1 was subjected to three organic extractions with one volume *n*-butanol, with the aid of a decantation funnel. The lipopeptide was dried, evaporating the organic phase with a vacuum concentrator, and dissolved in distilled water. This solution was used to hydrate the PDA powder. Then, pH was adjusted to 5.6 ± 0.2 and the medium was sterilized. Medium was prepared with final crude lipopeptide extract concentrations of 10 and 20 mg mL^−1^. After this, a 4-mm plug of *V. dahliae* mycelium was placed in the center of the plates, which were incubated at 25 °C for 20 days. The control consisted of PDA prepared with distilled water. The percentage of mycelium inhibition was calculated using the ImageJ software. Three replicates were conducted. 

The minimal inhibitory concentration (MIC) of the crude lipopeptide extract of strain XT1, defined as the lowest concentration that totally inhibits fungal growth, was also determined. Briefly, a mix of 900 µL of *V. dahliae* conidiospore suspension, obtained as explained above, and 300 µL of strain XT1 crude lipopeptide extract were placed in the wells of a 48-well culture plate. The final concentrations of lipopeptide in the wells were 0.5, 1, 2, 4, 6, 8, 10, and 20 mg mL^−1^. The same controls as in Section 2.3.1 were used. The growth of the *V. dahliae* was assessed visually after 30 days of incubation at 25 °C with 100-rpm rotary shaking. The experiment was carried out in triplicate. 

### 2.4. Preventive Activity by B. velezensis XT1 of Verticillium Wilt on Olive Trees 

*B. velezensis* XT1 was evaluated as a protective or preventive tool for the management of the *Verticillium* wilt disease on olive trees. Forty-eight young (~9-month old) olive trees (*O. europaea* cv. Picual) in 1.2-L pots filled with non-sterile potting soil (Compo Sana universal substrate, Compo, Münster, Germany) were used. The plants were divided in two separate replicates, each with 24 trees. They were placed randomly in a greenhouse in Andalusia (Spain) at 25/20 °C (day/night), relative humidity 60–80%, long-day photoperiod (16:8 h light:dark), luminosity of 250 μS cm^-2^ s^−1^, and drip irrigation systems (~50 mL per day). The experiment lasted 3 months. Briefly, 11 and 3 days before pathogen inoculation, plants were bacterized with *B. velezensis* XT1 as follows. The inoculation was conducted directly in the soil, at 2 cm from the plant stem, with 1 mL of a 5-day culture of strain XT1 in SG (10^9^ CFU mL^−1^). Then, following the methods of Colella et al. [54] and Trapero et al. [55], the plants were uprooted, washed in running water, and infected with a conidiospore suspension of *V. dahliae* by root immersion for 10 min, and later, changed to new pots with fresh non-sterile potting soil. The conidiospore suspension of *V. dahliae* was obtained with a 7-day culture of the fungus*,* which was crushed and filtered through an 80-µm pore gauze and adjusted to a concentration of 10^7^ conidiospores mL^−1^. Three and 11 days after the infection, the group of treated plants was inoculated again with *B. velezensis* XT1 as explained above. Control of each replicate consisted of 12 infected non-treated olive trees. 

The presence of *V. dahliae* on olive tree leaves was determined by PCR 30 and 90 days after fungal infection. Briefly, total DNA from 5 g of a pool of ~30 olive tree leaves of each condition and replicate was purified using the methodology described by Doyle and Doyle [56]. Detection of *Verticillium* was done using the specific primers DB19Fwd 5′-CGGTGACATAATACTGAGAG-3′ and DB22Rev 5′-GACGATGCGGATTGAACGAA-3′, which amplify specific polymorphic DNA bands of 539-bp in defoliating *V. dahliae* isolates [57]. 

Polyphenol oxidase (PPO) activity was measured to evaluate the stress caused by the infection 30 days after the inoculation of the plants with *V. dahliae*. Briefly, 5 g of a pool of 30 olive tree leaves of each condition and replicate were homogenized with 0.6 g of polyvinylpolypyrrolidone and 20 mL of a buffer solution containing NaH_2_PO_4_×2H_2_O 0.07% (*w*/*v*) and 1.6% (*w*/*v*) Na_2_HPO_4_×12H_2_O. After 2 min, the mixture was filtered through paper and centrifuged at 10,000×g for 15 min at 4 °C. One-hundred μL of the supernatant were combined with 2.8 mL phosphate buffer 0.2 mM pH 7 and 50 μL catechol 60 mM. The mix was maintained at 25 °C and the variation in absorbance was measured at 420 nm for 3 min. PPO activity was expressed as the change in the absorbance unit per second per gram of plant fresh weight [58]. Leaves of infected non-treated olive trees were used as the control.

The degree of plant infection and the number of microsclerotia in the soil were determined 90 days after the inoculation with *V. dahliae*. The first was assessed determining the incidence rate and the percentage of severity. The incidence rate referred to the percentage of plants visually affected (by chlorosis, leaf and shoot necrosis or defoliation) in each treatment. The percentage of severity, measured as the relative area under the disease progress curve (AUDPC), was determined according to López-Escudero et al. [59]. Regarding the number of microsclerotia in the soil, this was assessed according to Kabir et al. [60]. Briefly, a sample of 5 g of soil of each pot was taken (~3 cm from the plant stem, at ~10-cm depth), homogenized with a mortar, and dried at 37 °C for 2 days. Then, the powder was transferred to a tube with 2.5 mL of a solution of 7.5 mg mL^−1^ methionine in order to break fungistasis and improve detection [61], and was incubated with an open lid at 30 °C for 3 days. The resulting dry powder was grinded up again and placed over Sorensen’s NP10 medium [60] supplemented with 50 µg mL^−1^ chloramphenicol and 10 µg mL^−1^ streptomycin. After incubation at 20 °C for 15 days in the absence of light, the surface of the medium was washed with sterile distilled water and the number of microsclerotia was quantified at 10X magnification. The control consisted of potting soil from infected non-treated olive trees.

### 2.5. Field Experiments with B. velezensis XT1 on Olive Trees Diseased by V. dahliae

*B. velezensis* XT1 was tested as a palliative treatment on twenty adult (~10-year old) Picual olive trees that showed symptoms of affectation by *V. dahliae*. Plant infection was confirmed by isolating the pathogen from affected areas on PDA, observation by optical microscopy after safranin staining [62], and determination of the number of microsclerotia in the soil (~15-cm depth) [60]. The trees were planted in the open field in a parcel in Granada, Spain (37°25’17.4”N 3°44’38.5”W). The type of soil in the parcel corresponds to calcaric regosol, according to FAO [63]. Treated trees were inoculated with 20 L of a 5-day culture of *B. velezensis* XT1 in MOLP medium (10^10^ CFU per olive tree), which was injected in the soil (~40-cm depth) in six points at ~80 cm from the olive tree trunk. Ten diseased non-treated trees were used as control. Seven months after the inoculation, another application of *B. velezensis* XT1 was carried out, as explained before. Fourteen months after the first inoculation, the symptom severity of the olive trees was assessed using a 0–4 rating scale according to the percentage of plant tissue affected, being 0 = healthy plant or plant without symptoms, 1 = affected plant in 1–33%, 2 = 34–66%, 3 = 67–99%, and 4 = dead plant [59]. 

### 2.6. Permanence of B. velezensis XT1 in Soil

To study the permanence of the *B. velezensis* XT1 population in the soil after plant inoculation, a rifampicin-resistant (Rif^R^) XT1 derivative was used to facilitate selection. Briefly, to obtain this derivative, 5 mL of an overnight culture of strain XT1 in TSB (10^8^ CFU mL^−1^) were centrifuged at 5,000× g for 10 min. After washing twice with 0.8% NaCl (*w*/*v*), the pellet was dissolved in 400 μL of the same saline solution. Then, 100 μL were plated in each plate TSA supplemented with 100 µg mL^−1^ rifampicin. After incubation at 30 °C for 3 days, individual spontaneous rifampicin-resistant mutant colonies were selected.

For the assay, twenty 6-month old Picual olive trees were used. Trees were divided in two separate replicates, each with 10 plants. The olive trees were in 1.6-L pots with Compo Sana universal substrate (Compo, Münster, Germany). They were placed randomly in a greenhouse at 25/20 °C (day/night), relative humidity 60–80%, long-day photoperiod (16:8 h light:dark), and luminosity of 250 μS cm^−2^ s^−1^. Then, *B. velezensis* XT1 Rif^R^ was cultured in MOLP for 2 days (10^8^ CFU mL^−1^) and applied directly in the soil (10^6^ UFC g^−1^ of substrate) of half the plants in each replicate. The control of each replicate consisted of five non-treated olive trees. All plants were watered every 24 h with a similar amount of water (~50 mL) through a drip irrigation system. Bacterial count was determined 30, 60, 90, and 120 days after the experiment started. In brief, soil was homogenized with 0.8% (*w*/*v*) NaCl, and 10-fold serial dilutions were prepared and plated on nutrient agar (NA) supplemented with 100 µg mL^−1^ rifampicin and incubated for 48 h at 30 °C in order to estimate XT1 Rif^R^ CFU g^−1^. 

### 2.7. Ability of B. velezensis XT1 to Colonize Endophytically Olive Roots

The capacity of *B. velezensis* XT1 to grow endophytically in olive roots was evaluated as follows, using the olive tree plants of the previous experiment. After 120 days, the plants were extracted from the pots and the soil was discarded. The roots were cut and washed with sterile distilled water and ethanol 70% (*v*/*v*). Then, they were submerged in NaClO 5% (*v*/*v*) for 1.5 min. After vortexing, the NaClO was discarded and the roots were washed three times with abundant sterile distilled water. Finally, they were cut into small pieces (~1 cm) and homogenized with a mortar and pestle in 5 mL 0.8% (*w*/*v*) NaCl. The control of each replicate consisted of roots from five non-treated olive trees. Bacterial count was determined on NA supplemented with 100 µg mL^−1^ rifampicin as previously explained. 

### 2.8. Statistical Analyses

Statistical analyses were conducted using the R software [64]. Differences with the control in the experiments conducted were assessed by post hoc Tukey analysis. A normal distribution for measured variables, as well as a quasi-Poisson distribution and square root link function for the counted variables, were assumed.

## 3. Results

### 3.1. Biosafety of B. velezensis XT1

**Figure 1:**
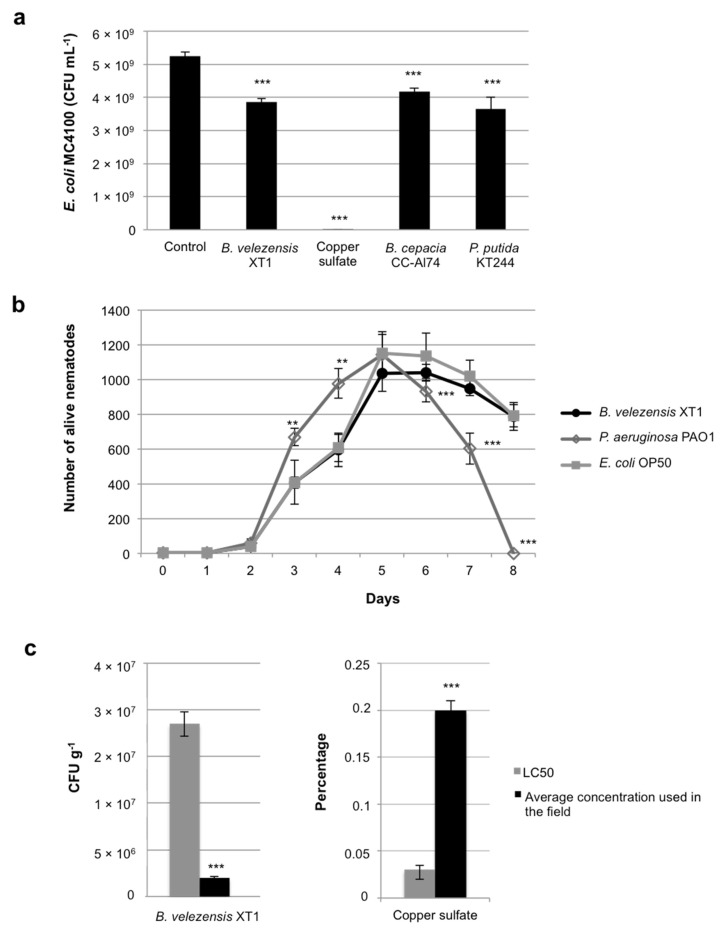
Biosafety evaluation of *Bacillus velezensis* XT1. (**a**) *Escherichia coli* MC4100 sensitivity assay. *Pseudomonas putida* KT2440, *Burkholderia cepacia* CC-Al74 and 0.2% (*w*/*v*) copper sulfate were used for comparison. Non-inoculated tryptic soy broth (TSB) was used as a control. (**b**) *Caenorhabditis elegans* N2 pathogenicity test. *Pseudomonas aeruginosa* PAO1 was used for comparison. *E. coli* OP50 was used as a control. (**c**) Comparison of LC50 values obtained in *C. elegans* N2 and average concentrations used in the field. Copper sulfate was used for comparison. Significant differences between the LC50 and the field concentration are indicated by asterisks (** *p* < 0.01, ***, *p* < 0.001).

The safety of *B. velezensis* XT1 was evaluated using three types of tests. In the sensitivity assay, strain XT1 reduced the population of *E. coli* MC4100 (Figure 1a). This reduction, however, was significantly (*p* < 0.05) inferior to that of copper sulfate, usually used as a fungicide in agriculture, which completely inhibited the growth of *E. coli* MC4100 (Figure 1a). In the Microtox test, the impact of *B. velezensis* XT1 on the metabolism (luminescence) of *A. fischeri* NRRL B-11177 was evaluated. No significant reduction in luminescence was observed after 10 min of exposition. After 15 min, the EC50 of strain XT1 was reached with a solution in distilled water, with 90% (*v*/*v*) of culture supernatant. Finally, the toxicity of *B. velezensis* XT1 was assessed using the nematode *C. elegans* N2. After 8 days of incubation, there were not significant differences between strain XT1 and the control *E. coli* OP50. The results obtained for *P. aeruginosa* PAO1, which caused a high mortality of the nematodes, were significantly different (*p* < 0.01) to the results obtained by strain XT1 and *E. coli* OP50 (Figure 1b). Regarding the LC50 value of *B. velezensis* XT1, it was found that the concentration used in the field as being effective (2×10^6^ CFU g^−1^ soil) is nearly ten times smaller than its LC50, identified to be 1.8×10^7^ CFU g^−1^. In the case of the chemical fungicide of agricultural use, copper sulfate, its average effective dose, 0.2% (*w*/*v*), was higher than its respective LC50, 0.03% (*w*/*v*) (Figure 1c). In both cases, differences between LC50 and average concentration used in the field were found significant (*p* < 0.001). Mortality of nematodes was not observed in the non-treated control, and therefore a LC50 value was not calculated.

### 3.2. In Vitro Antifungal Activity of B. Velezensis XT1 

**Figure 2:**
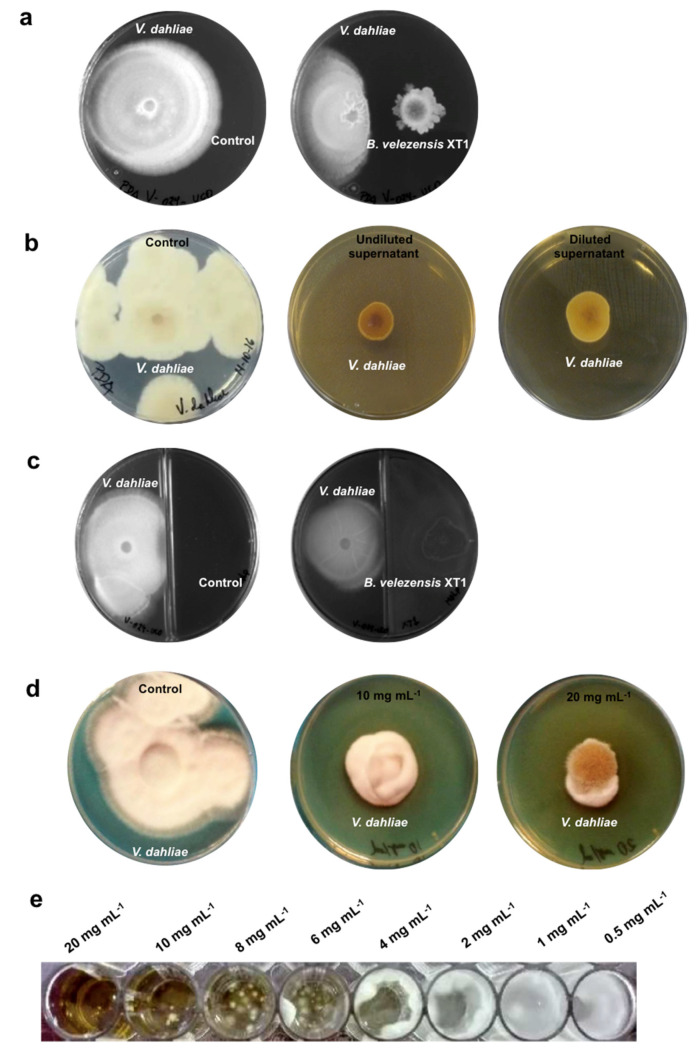
In vitro antifungal activity of *Bacillus velezensis* XT1 against *Verticillium dahliae* V024. (**a**) Assay on solid media with the whole culture of strain XT1. Non-inoculated tryptic soy broth (TSB) was used as a control. (**b**) Assay on modified medium prepared with the half-diluted and undiluted supernatant of strain XT1. Control consisted of medium hydrated with distilled water. (**c**) Volatile compounds antifungal test. Control consisted of non-inoculated TSB. (**d**) Crude lipopeptide extract antifungal test. Control consisted of medium hydrated with distilled water. (**e**) Minimal inhibitory concentration (MIC) of the crude lipopeptide extract determined in a liquid assay.

The antifungal activity of *B. velezensis* XT1 against *V. dahliae* V024 was assessed in several types of experiments. In the liquid media assay, the supernatant of strain XT1 completely abolished (100% inhibition) conidiospore germination and, therefore, later mycelium formation of *V. dahliae* after 30 days of incubation. The plate assay demonstrated that *B. velezensis* XT1 had an in vitro antagonistic effect and reduced by over 56% the mycelia of *V. dahliae* after 20 days of incubation (Figure 2a). In the modified plate assay using the undiluted and half-diluted supernatant of strain XT1 liquid culture, *Verticillium* mycelium was reduced by 94 and 91%, respectively (Figure 2b). The volatile compounds of strain XT1 reduced the growth of *V. dahliae* by 34% (Figure 2c). Regarding the crude lipopeptide extract, it reduced the growth of the fungi by 80% and by 88% when used at a concentration of 10 and 20 mg mL^−1^, respectively (Figure 2d). In liquid medium, the MIC of the crude lipopeptide extract was determined to be at 20 mg mL^−1^ (Figure 2e). In all the assays, the differences with the respective controls were significantly different (*p* < 0.05).

### 3.3. Prevention of Verticillium Wilt on Olive Trees Treated with B. velezensis XT1

**Figure 3:**
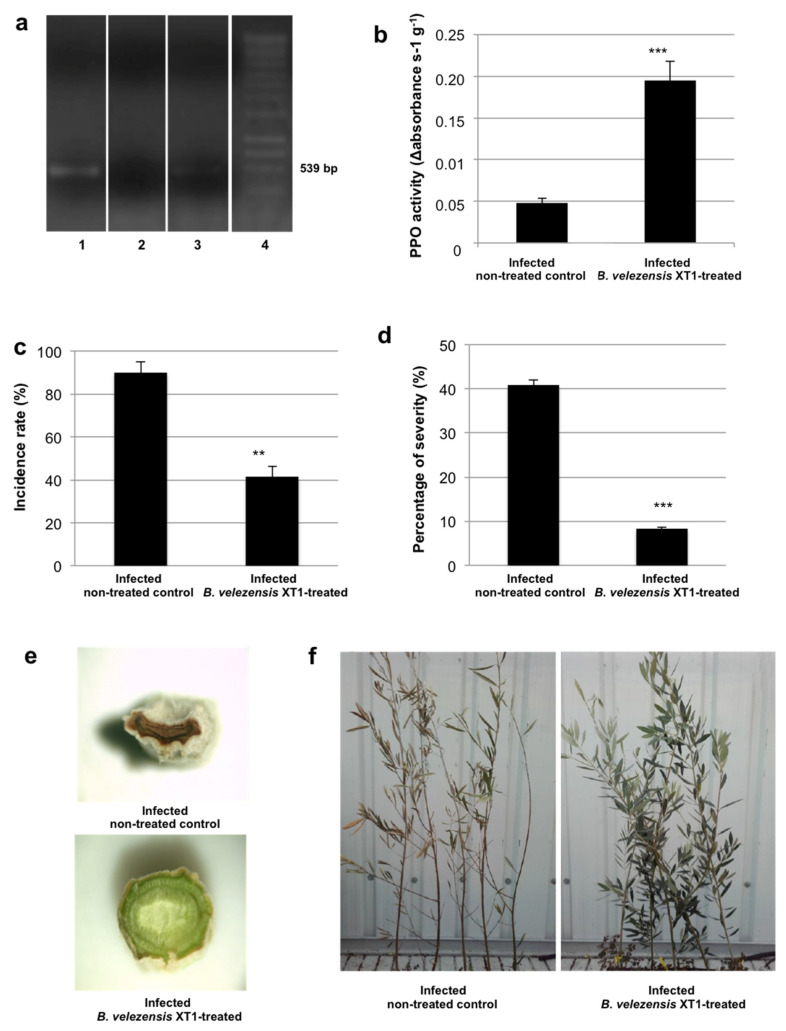
Prevention of *Verticillium* wilt on young olive trees by *Bacillus velezensis* XT1. (**a**) PCR amplification of *Verticillium dahliae* in olive tree leaves. 1: Infected non-treated control, 2: infected *B. velezensis* XT1-treated plants 30 days after infection, 3: infected *B. velezensis* XT1-treated plants 90 days after infection, 4: molecular weight ladder. (**b**) Polyphenol oxidase (PPO) activity. Leaves of infected non-treated olive trees were used as control. (**c**) Incidence rate of infected non-treated control and infected XT1-treated olive trees. (**d**) Percentage of severity of infected non-treated control and infected XT1-treated olive trees. (**e**) Transversal cut of infected non-treated control and infected XT1-treated olive tree branches. (**f**) Whole plant aspect of infected non-treated control and infected XT1-treated trees. Significant differences with the control are indicated by asterisks (**, *p* < 0.01; ***, *p* < 0.001).

The effect of the preventive treatment by *B. velezensis* XT1 on olive trees artificially infected with *V. dahliae* was estimated using young trees (~9-month old). Thirty days after the infection of the olive trees with *V. dahliae* (i.e., 45 days after the first inoculation with strain XT1), no disease symptoms could be observed. The presence of the fungi was detected by PCR in the non XT1-treated olives, although no amplification was obtained in the olive trees which had been inoculated with *B. velezensis* XT1 (Figure 3a). Ninety days after the infection with *V. dahliae*, the fungus was detected by PCR also in the XT1-treated olives, although with a weak amplification (Figure 3a). 

At the end of the experiment, the number of microsclerotia in the soil was significantly (*p* ≤ 0.05) diminished by 20.2% due to the application of *B. velezensis* XT1, more exactly, from 47.2 to 36.4 microsclerotia g^−1^ soil. PPO activity, measured to evaluate plant response to stress, was increased by 395% (*p* < 0.05) in the XT1-treated plants in comparison to the control (Figure 3b).

Regarding the degree of infection of the trees, this was assessed determining the incidence rate and percentage of severity. The results show a significant (*p* < 0.05) reduction of 54% in the incidence rate (Figure 3c) and of ~80% in the percentage of severity (Figure 3d) in the infected XT1-treated olive trees in comparison with the infected non-treated control trees. The differences could easily be observed at the branch (Figure 3e) and whole plant level (Figure 3f). 

### 3.4. Reduction in Verticillium Wilt by B. velezensis XT1 on Diseased Olive Trees 

**Figure 4:**
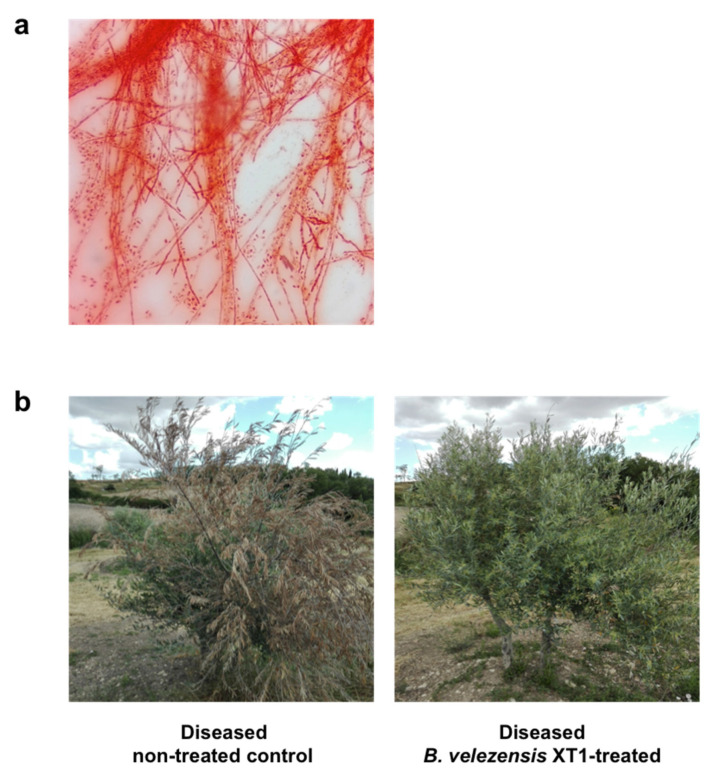
Palliative treatment of *Verticillium* wilt on diseased adult olive trees by *Bacillus velezensis* XT1. (**a**) Microscopic observation of *V. dahliae* isolated from affected trees. (**b**) Olive trees in the field experiment before and after the treatment with strain XT1. Control consisted of diseased non-treated trees.

The effect of the palliative treatment with *B. velezensis* XT1 on adult (~10-year old) diseased olive trees was evaluated in a field experiment in a naturally infected soil. Plant infection by *V. dahliae* was confirmed by isolating the fungi from affected areas on PDA and by microscopic observation (Figure 4a). The number of microsclerotia of *Verticillium* was determined to be 300 microsclerotia g^−1^ soil. At the beginning of the assay, symptom severity of the olive trees was ~1.6, according to the 0–4 rating scale. Fourteen months after the treatment with *B. velezensis* XT1, symptom severity was significantly (*p* < 0.05) reduced by ~63% in the olive trees treated with strain XT1 (Table 1). Differences could easily be assessed by visual inspection (Figure 4b). 

### 3.5. Permanence of B. velezensis XT1 in Soil

The permanence of *B. velezensis* XT1 in the soil after being inoculated in the olive trees was measured using a Rif^R^ XT1 strain. The results showed that the population of strain XT1 was maintained at an average count of 3.5×10^5^ CFU g^−1^ of soil until 30 days after the inoculation, when it decreased to 5.9×10^4^ CFU g^−1^ and was kept stable until the end of the experiment, 120 days later. No Rif^R^ colonies could be recovered from the soil of the non-treated control olive trees. 

### 3.6. Endophytic Growth of B. velezensis XT1 in Olive Roots

The ability of *B. velezensis* XT1 to grow endophytically on olive roots was tested using a Rif^R^ XT1 strain. One-hundred and twenty days after the inoculation of the olive trees with strain XT1, the average number of endophytic XT1 was reported as 3.5×10^4^ CFU g^−1^ of fresh root. No Rif^R^ clones were recovered in the non-treated control.

## 4. Discussion

In the agriculture sector, *Verticillium* wilt is among the most devastating fungal diseases worldwide. It affects hundreds of different plant species, including high value agricultural crops such as olive trees [6]. A major issue that concerns *Verticillium* disease management [31] is increasing soil salinization. In recent years, novel eco-friendly strategies are being sought in order to control plant diseases and stimulate plant growth in such a soil salinity scenario [65], with the aim of rendering the agriculture industry more sustainable and keeping a healthy environment. 

*B. velezensis* XT1, previously isolated from a rhizosphere sample taken from a saline soil [0.6% (*w*/*v*) NaCl], is a salt-tolerant biocontrol strain that has the ability to grow in a wide range of salt concentrations, from 0 to 12% (*w*/*v*) NaCl [34]. Strain XT1 has been described to promote plant growth due to different metabolic features such as nitrogen-fixation, siderophore production or synthesis of enzymes [34]. One of these enzymes, the 1-aminocyclopropane-1-carboxylate (ACC) deaminase, is involved in increased disease tolerance against *V. dahliae* [66]. Despite the fact that some strains of the *B. velezensis* species have recently been described as biocontrol agents against *Verticillium* wilt on olive trees, such as *B. velezensis* OEE1 [30], the activity of different isolates may differ due to the existence of strain-specific clusters of genes, and consequently, due to the synthesis of special enzymes and metabolites which are involved in both pathogen suppression, growth promotion, and salt tolerance. Regarding the latter, we believe that this is a desirable trait in *Verticillium* biocontrol agents, but future experiments are needed to assess the effect of the salt-tolerant strain XT1 on *V. dahliae* under saline conditions. Soil salinity is an upcoming problem due to intensive agriculture and climate change, especially in Andalusia, South Spain [67], the world’s largest producer of olive oil in the world [9]. According to Kaurichev [68], soils containing more than 0.2% (*w*/*v*) soluble salts should be considered as saline soils. Although olive trees are considered moderately tolerant to salinity [69], this abiotic stress leads to an increase in plant colonization and disease severity by *V. dahliae* [31,32] and affects the antagonist activity of some *Verticillium* biocontrol agents [33]. 

In this study, *B. velezensis* XT1 showed strong antifungal activity against the highly virulent defoliating *V. dahliae* V024. The activity in vitro was higher in liquid than in solid medium, which has also been observed against other phytopathogenic fungi, such as *Botrytis cinerea* [34,49]. Similar inhibition rates against *V. dahliae* were observed in plate assays with *B. thuringiensis* isolates [70] and a non-pathogenic strain of *Fusarium oxysporum* [25]. Regarding the antifungal activity of the supernatant, this indicates that the activity is due to extracellular metabolites and is not only contact-dependent. In this work, the undiluted and half-diluted supernatant of *B. velezensis* XT1 showed a strong antifungal activity against *V. dahliae*. For instance, the half-diluted supernatant reduced fungal mycelium by 91%, while Azabou et al. [30] described that the half-diluted supernatant of *B. velezensis* OEE1 had an activity of 69%. Some of the extracellular metabolites produced by bacteria are lipopeptides, biosurfactant molecules with activity against numerous plant pathogens [21], and volatile organic compounds [20]. Lipopeptides, apart from having antifungal activity, may be involved in inhibiting microsclerotia formation in *V. dahliae*, according to Yu et al. [71]. Strain XT1 was previously described to produce large amounts of heat-stable lipopeptides, which were identified by electrospray quadrupole time-of-flight mass spectrometry as four different types of surfactin, three bacillomycins D (iturin family), and fengycin A and B [49]. The exact composition of the crude lipopeptide extract of strain XT1 was not quantified, although in other *B. velezensis* strains, it was described to be ~40 µg mL^−1^ of surfactin, ~30 µg mL^−1^ of iturin, and ~140 µg mL^−1^ of fengycin [72]. In the case of *B. velezensis* OEE1, some lipopeptides have been identified by PCR amplification [30], although such detection method using specific primers does not imply production of the corresponding lipopeptide, as observed with iturin in strain XT1 [49]. In this study, the crude lipopeptide extract of strain XT1 reduced fungal growth by more than 80% at different concentrations. However, there are no data available regarding the activity of lipopeptide biosurfactants for *B. velezensis* OEE1 [30]. With regard to volatile compounds, they are well known for having antifungal activity, plant growth promoting activity, and inducing systemic resistance [73,74,75]. The volatile organic compounds of *B. velezensis* XT1 inhibited by 34% mycelium growth of *V. dahliae*, which is comparable to the 40% inhibition reported for *B. velezensis* OEE1 [30], although the experimental set up is not exactly the same. Further studies need to be conducted to characterize such volatile compounds in strain XT1.

An essential requisite for a microorganism to be applied in the field should be its biosafety [76]. Although many bacterial strains have been proposed as biocontrol and/or plant growth stimulants in the last decade, their toxicity has not been tested. In this study, we have evaluated the biosafety of *B. velezensis* XT1 using a series of experiments proposed by Vílchez et al. [37]. Two tests were used to assess the impact on other microbial communities. In the test on *E. coli* MC4100, strain XT1 showed an antagonistic effect, although its effect was lower in comparison to the biocontrol *B. cepacia* CC-Al74 and to copper sulfate, used as a fungicide in agriculture settings. In the test Microtox assay, the supernatant of strain XT1 did not reduce the luminescence of *A. fischeri* NRRL B-11177, and the EC50 was reached with a solution in distilled water with 90% (*v*/*v*) of culture supernatant, meaning an absence of toxicity of strain XT1 [40]. Finally, the impact of XT1 on the survival of soil nematodes was evaluated with a *C. elegans* N2 bioassay. It was observed that *B. velezensis* XT1 did not have a negative effect on the viability of the nematodes. Additionally, it was found that the effective concentration of XT1 used in the field was nine times smaller than its LC50, meaning very low toxicity. However, in the case of the chemical fungicide of frequent agricultural use, copper sulfate, its effective dose was superior to its LC50. All these data, together with the fact that the majority of environmental *Bacillus* species (such as *B. subtilis* and *B. amyloliquefaciens*) belong to biosafety level 1 category, reflect that strain XT1 is not toxic and can be used for formulation development and field scale applications [76]. Regarding the other *B. velezensis* strain with activity against *V. dahliae*, strain OEE1, there are no available data concerning its biosafety, and a toxic and harmful effect is observed in plants when the inoculum of the strain is 10^6^ CFU g^−1^ [30].

Although not a requisite, some desirable characteristics of biological control agents are plant growth promotion, the ability to deal with adverse environmental conditions, persistence in soil for long periods, and the capacity to colonize endophytically plant roots. In a previous work, we showed that *B. velezensis* XT1 promoted growth of different plant species [34]. Moreover, XT1 has shown to be highly resistant to adverse environmental conditions such as salinity, temperature or pH, and to produce spores [34], which allow bacterial survival for long periods in stress conditions [77]. Regarding the persistence in soil, the rifampicin-resistant derivative of strain XT1 was found at high density several months after inoculation. Our results agree with the data obtained by Young [78] on *B. cereus* persistence. No data regarding the monitoring of the permanence of the inoculum on soil were published for *B. velezensis* OEE1 [30]. With respect to the ability to effectively colonize endophytically plant roots, it could play an important role in the biocontrol effectiveness of strain XT1, which was also observed in *B. velezensis* OEE1 [30] and in *P. fluorescens* PICF7 [79]. However, these results must be interpreted cautiously, since a Rif^R^ XT1 strain was used instead of the wild type. Rifampicin resistance is commonly used as a reliable marker to study plant colonization by *Bacillus* spp. and other soil bacteria [80,81,82,83,84]. Despite the fact that pleiotropic effects have recently been observed in a *Bacillus velezensis* rifampicin-resistant mutant strain [85], population dynamics of wild-type and antibiotic-resistant derivatives were observed to be similar in other *Bacillus* species [82].

Once the antifungal activity in vitro, biosafety, and other desirable traits of *B. velezensis* XT1 were determined, we evaluated its use as a preventive measure (treatment before infection occurs) and palliative action (application after infection) on olive trees infected with *V. dahliae*. For the preventive treatment, olive plants were infected using a root-dip inoculation method with *V. dahliae* conidiospores, as used by other authors [2,54,55]. However, alternative infection methods have recently been proposed, since root-dip inoculation make it difficult to assess the effect of biocontrol agents due to the high inoculum pressure of the pathogen and the fact that, once the pathogen has colonized the plants, it is highly inaccessible to biocontrol agents [35]. For both preventive and palliative treatments, the Picual variety was used for the experiments for being the most produced in Spain and one of the varieties most affected by *Verticillium* [86,87]. 

In the preventive treatment on young olive trees, *B. velezensis* XT1 reduced the percentage of severity by ~80%. This is very superior to the ~47% reduction obtained by *P. alvei* K165 [27] and similar to the results obtained with *B. velezensis* OEE1 [30]. However, the plant system used by the latter was seedlings grown in modified solid medium [88] during a short period, instead of olive trees in pots with soil substrate and maintained during long incubation periods. Additionally, it is not clear if the *Verticillum* isolate used by Azabou et al. [30] is highly virulent and defoliating, as is the case of *V. dahliae* V024, and the conidiospore suspension they use to infect their plants is only 10^4^ conidiospores mL^−1^, while in our study, it is of 10^7^ conidiospores mL^−1^. Regarding the incidence rate, strain XT1 reduced by 54% this parameter. This percentage of reduction is higher than the values obtained by Cabanás et al. [89], Mulero-Aparicio et al. [90], and Varo et al. [91] with *Pseudomonas* spp., *Fusarium oxysporum*, and *Bacillus* spp., respectively, on infected olive trees. Regarding the number of microsclerotia in the upper layer of the soil [92], it was reduced by ~20.2% after the addition of strain XT1. This leads to the belief that the antifungal activity of XT1 is more due to its interaction with the plant than from its activity in the soil. Varo et al. [91] obtained higher percentages of microsclerotia reduction, but the inoculum of the fungi and the *Bacillus* biocontrol organisms were considerably lower and higher, respectively. With regard to the detection of *V. dahliae* by PCR in the infected trees, the treatment with *B. velezensis* XT1 seemed to diminish the presence of the fungus in the plant. Although our PCR results are not quantitative, a reduction in the amount of PCR products could indicate a decrease in the pathogen within the infected plant, as observed by other authors [93,94]. In order to give a more accurate indication on the delay of infection and the quantification of *Verticillium* biomass within the plant tissue, this should be confirmed in further studies with quantitative techniques such as qPCR [95], even though these fail to discriminate between DNA from viable and dead fungal structures [96]. Regarding the evaluation of the stress caused by the infection on the olive trees, PPO activity is a parameter related with the content of phenolic compounds, which are metabolites that play different roles in plants, such as disease resistance [97]. If these compounds are not enough to stop pathogenic infection, they increase in the infection site and act as substrates to PPO enzymes, which catalyze the conversion of phenolic compounds into o-quinones, that are highly toxic for phytopathogens [98]. We found that the addition of strain XT1 increased PPO in comparison to the control, which indicates a higher resistance towards the infection [99]. Recently, some *Streptomyces* isolates showed the ability to increase PPO in leaves of cotton plants inoculated with *V. dahliae* [100], although the parameters were measured only three days after infection in plants cultured in vitro, and not in vivo as in our study. Unfortunately, the plant response to stress caused by the infection was not described in the other *B. velezensis* strains used against *Verticillium* wilt, such as strain OEE1, and therefore, comparison of the results is not possible [30].

Ultimately, the antifungal activity of *B. velezensis* XT1 was evaluated in the field as a palliative measure on adult olive trees that showed *Verticillium* wilt symptoms (symptom severity 1.6). The presence of *V. dahliae* was determined by the count of microsclerotia in the soil, the isolation of the fungi from diseased plant tissues, and the observation by microscopy of the fungi typical features. The palliative treatment with strain XT1 considerably reduced the severity of the symptoms by ~63%. Management of *Verticillium* wilt in woody long-lived plants has been investigated to a reduced extent. To date, biological control experiments on olive trees are very scarce. Azabou et al. [30] recently conducted a field experiment in a naturally infected soil with a content of ~98 microsclerotia g^−1^ soil, while in our case, there were 300 microsclerotia g^−1^ soil, but the experimental set up is not clearly explained to allow comparison. In addition, Mulero-Aparicio et al. [90] have recently tested several biocontrol agents under field conditions. Although the characteristics of the experiments are not the same as in our study, some comparison can be performed. For instance, *P. fluorescens* PICF04 did not reduce symptom severity of infected adult olive trees, while a mix of bacterial species (*Rhodopseudomonas palustris*, *Rhodobacter sphacrodes*, *Lactobacillus plantarum*, *L. casei*, *Streptococcus lactis*, *Saccharomyces* spp., and *Streptomyces* spp.) only reduced the disease severity by 30%. These results are lower than those observed in our study with *B. velezensis* XT1. Nevertheless, more studies need to be conducted in the future to this end in order to evaluate if the initial symptom severity state of the diseased trees could have an impact on the effect with strain XT1.

## 5. Conclusions

The salt-tolerant *B. velezensis* XT1 shows strong antifungal activity against the highly virulent defoliating pathogen *V. dahliae* V024, which was demonstrated in vitro as well as on olive trees in greenhouse and field experiments with both preventive and palliative treatments. This, in addition to other characteristics of strain XT1 such as biosafety, enhancement of plant response to stress, persistence in soil, endophytic colonization of olive tree roots, and reduction of fungal microsclerotia in soil make it a promising biocontrol agent to be used against *Verticillium* wilt in the agriculture industry. 

## Figures and Tables

**Table 1:** Symptom Severity in the Palliative Treatment of *Verticillium* Wilt on Adult Diseased Trees with *B. velezensis* XT1.

Treatment	Before the Treatment	After 14 Months
Control	1.6 ± 0.6^a^	2.7 ± 1.2 ^a^
*B. velezensis* XT1	1.6 ± 0.6^a^	1.0 ± 0.9 ^b^

Mean values within a column followed by different lowercase letters (^a^,^b^) indicate that they are significantly different (*p* ≤ 0.05).
